# Etched Polymer Fibre Bragg Gratings and Their Biomedical Sensing Applications

**DOI:** 10.3390/s17102336

**Published:** 2017-10-13

**Authors:** Ginu Rajan, Kishore Bhowmik, Jiangtao Xi, Gang-Ding Peng

**Affiliations:** 1 School of Electrical, Computer and Telecommunications Engineering, University of Wollongong, Wollongong 2522, Australia; jiangtao@uow.edu.au; 2 HFC Network Assurance, HFC Operations, NBN, Sydney 2060, Australia; kishorebhowmik@nbnco.com.au; 3 School of Electrical Engineering and Telecommunications, UNSW Australia, Sydney 2052, Australia; g.peng@unsw.edu.au

**Keywords:** polymer fibre Bragg grating, etched single mode fibre, biomedical sensing, pressure transducer, breathing rate monitor

## Abstract

Bragg gratings in etched polymer fibres and their unique properties and characteristics are discussed in this paper. Due to the change in material and mechanical properties of the polymer fibre through etching, Bragg gratings inscribed in such fibres show high reflectivity and enhanced intrinsic sensitivity towards strain, temperature, and pressure. The short-term and long-term stability of the gratings and the effect of hysteresis on the dynamic characteristics are also discussed. The unique properties and enhanced intrinsic sensitivity of etched polymer fibre Bragg grating are ideal for the development of high-sensitivity sensors for biomedical applications. To demonstrate their biomedical sensing capabilities, a high-sensitivity pressure transducer that operates in the blood pressure range, and a breathing rate monitoring device are developed and presented.

## 1. Introduction

In recent years, polymer fibre Bragg gratings (PFBGs) have gained much interest among researchers due to their unique advantages compared to their silica counterparts, such as inherent fracture resistance, high flexibility, large strain measurement range, and biocompatibility [1,2]. Advancements in the fabrication of single mode polymer optical fibres (SMPOFs) have enhanced the research and development of Bragg grating sensors in polymer optical fibres (POFs) [3,4] and considerable efforts in adopting the sensing capabilities of PFBGs are ongoing. Progress in SMPOF fabrication and enhanced photosensitivity have led to reduced inscription time, with grating inscription reported in minutes [5]. Though in the recent years the research work on polymer FBGs has been accelerated, it still lags behind that of silica FBGs.

Recent research on PFBG fabrication and sensing includes fabrication of polymer micro-fibre gratings, and applications of PFBG sensors in humidity sensing, force/pressure sensing, accelerometers, and for composite materials [6,7,8,9]. These research outputs give an indication that PFBG sensing is moving towards application-oriented research in niche areas. Difficulties in fabricating solid-core single-mode polymer fibres, high transmission loss, and coupling and standardization issues have hampered growth in this research area. However, the advantages of PFBGs, such as the low Young’s modulus, high temperature sensitivity, and large strain range, can find applications in areas such as biomedical sensing.

The recent research conducted by the authors demonstrated that fibre diameter reduction via solvent etching can change the material/mechanical properties of the polymer fibre such as Young’s modulus, Poisson’s ratio and the thermal expansion coefficient [10,11]. This knowledge was very crucial and led to further research in this area, as this is a unique way to intrinsically increase the sensitivity of sensors based on polymer fibres. Though there may be many techniques available to enhance the sensitivity of FBG sensors, extrinsically they increase size and involve further physical complexities [12,13]. Therefore, methods to improve the sensitivity intrinsically will be advantageous compared to any extrinsic methods. Intrinsic high-sensitivity sensors can be less complex and miniature in size compared to extrinsic ones and can provide solutions for important sectors, for example in biomedical/biomechanical applications, where there are needs that are still not addressed by existing sensors.

In this paper, an overview of the fabrication of etched polymer fibre Bragg gratings through solvent etching method and their stability are discussed. The enhanced intrinsic sensitivity of the sensor towards various physical parameters such as temperature, static and dynamic strain, and pressure that can lead to the development of biomedical sensors is also discussed. Due to the viscoelastic nature of polymer materials, POFs exhibit hysteresis and this limits the dynamic performance of the fibre. In this paper, the effect of hysteresis on the dynamic characteristics of PFBG is also studied. To further demonstrate the capability of the developed sensor in biomedical applications, a high-sensitivity blood pressure transducer and a breathing rate monitoring device based on the etched PFBG are developed and reported.

## 2. Etched Polymer Fibre Fabrication via Solvent Etching and Modification of the Fibre Properties

In a polymer fibre there will be a gradation of stress levels and microstructure of the polymer chains through the fibre cross section. Surface processing techniques such as etching of the fibre surface will expose the material, causing different stress levels and microstructure compared to the original, and as such result in different performance characteristics. Recently, the authors have observed that the sensitivity of PFBGs can be intrinsically enhanced through solvent etching [10,11] as it leads to a useful change in their material and mechanical properties, a reduction in the Young’s modulus (E), and an increase in the thermal expansion coefficient (TEC). As most of the physical measurements are directly or indirectly related to E and TEC, this phenomenon provides an advantage to polymer fibre grating devices towards their intrinsic sensitivity to various physical measurands compared to silica based ones. 

The single mode polymer fibre used in this work was fabricated from a core monomer mixture of Poly(methyl methacrylate) (PMMA )doped with Benzyl methacrylate (BzMA) in order to meet the requirements of the refractive index, while the cladding is of PMMA without dopants [2,3]. The core diameter of the fibre measured 12 µm, and fibres with two cladding diameters of ~180 ± 5 µm and 250 ± 5 µm were used in this study. This was to demonstrate that etching and not the diameter reduction changed the material and mechanical properties of the fibre and thus their intrinsic sensitivities. The fibres were single mode at the wavelength of operation between 1520 and 1570 nm.

**Figure 1:**
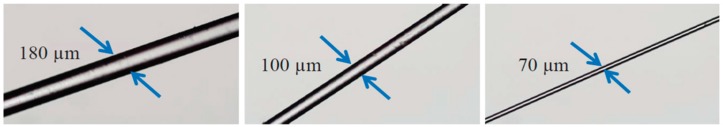
Microscopic images of polymer fibres before etching (180 µm) and after etching (100 µm and 70 µm).

To etch the fibre, a solvent etching method was adopted where a 1:1 combination of acetone and methanol was used. Acetone is a good solvent for polymer fibre, while methanol is added to control the rate of etching. A 1:1 ratio provides well-controlled etching, and an etched diameter accuracy of ±1 µm was achieved. A microscopic image of polymer fibre before and after etching is shown in Figure 1.

## 3. Bragg Gratings in Etched Polymer Fibres—Fabrication, Reflectivity and Stability

### 3.1. Fabrication and Reflectivity

**Figure 2:**
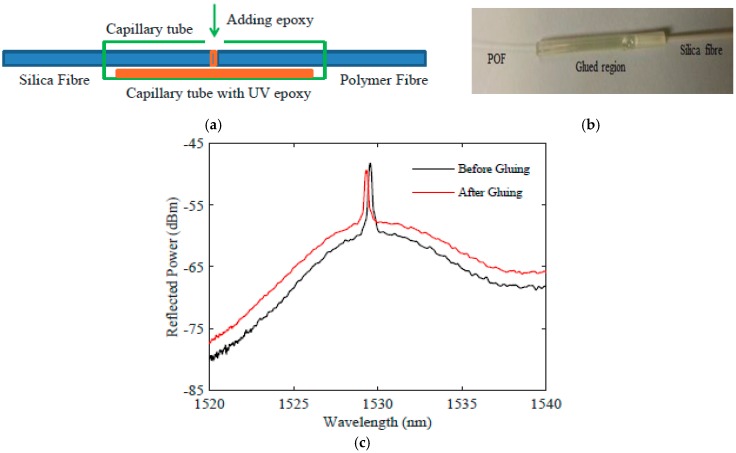
Polymer fibre Bragg gratings (PFBG) pigtail fabrication. (**a**) Schematic of the procedure; (**b**) the glued region of a PFBG pigtail; (**c**) response showing the impact of the epoxy on the PFBG reflection spectrum. POF: polymer optical fibre.

Bragg gratings in unetched and etched single-mode polymer fibres were fabricated by a standard phase-mask technique using a 50-mW Kimmon IK series He-Cd laser emitting light at 325 nm [14]. The phase mask was 10 mm long and was suitable for a 320-nm wavelength; it can produce gratings with a peak reflected wavelength of circa 1530 nm. To observe the Bragg grating reflection and transmission spectrum of the grating, a high-power broadband source was used and the spectra were monitored using an optical spectrum analyser. Due to the high transmission loss of the polymer fibre in the wavelength of operation (1530 nm), the length of the polymer fibre was limited to 10 cm and is connected (glued) to a silica fibre pigtail. The schematic of pigtail fabrication process is shown in Figure 2a where the polymer fibre and silica fibre are inserted into a 3-mm-diameter, 3-cm-long plastic capillary tube with a tiny hole in the middle to add UV light curable epoxy. The fibres are coupled using coupling stations and then the epoxy is added and cured while monitoring the light output to ensure that curing-induced epoxy shrinkage has not affected the coupling. The cured coupling region is shown in Figure 2b. The impact of gluing process on the typical PFBG spectrum can be seen in Figure 2c, where the signal to noise ratio was reduced for the glued one compared to the free space coupled one. This is due to difference in the refractive index of the epoxy and the fibres.

**Figure 3:**
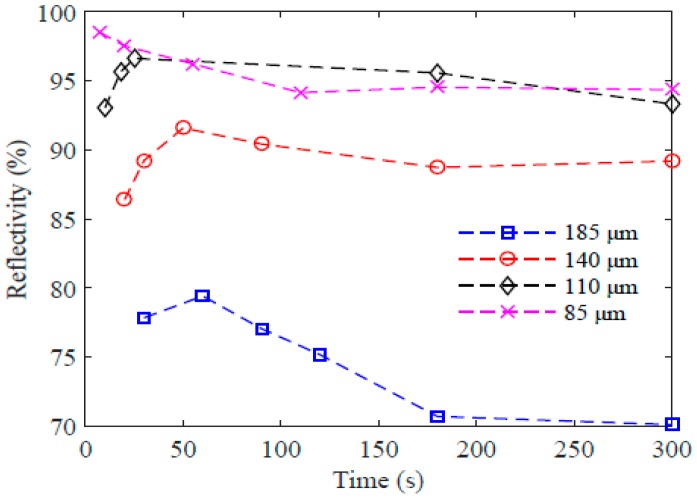
Reflectivity of the PFBGs with different diameters at various exposure times.

The measured reflectivity of the gratings inscribed in the etched fibres is shown in Figure 3, where a PFBG with a diameter of 85 µm shows the highest reflectivity, at 98.5% after just 7 s of exposure, whereas the reflectivity was reduced to 95% after 300 s of exposure. From the figure it can also be seen that the reflectivity can be improved by reducing the fibre diameter, and the time required to achieve the peak reflectivity also decreases with reduction in the fibre diameter. For the 85-µm fibre, peak reflectivity is obtained at 7 s, whereas for the unetched fibre of 185 µm an exposure time of 60 s was required to obtain the highest reflectivity of 79.42%.

### 3.2. Stability

**Figure 4:**
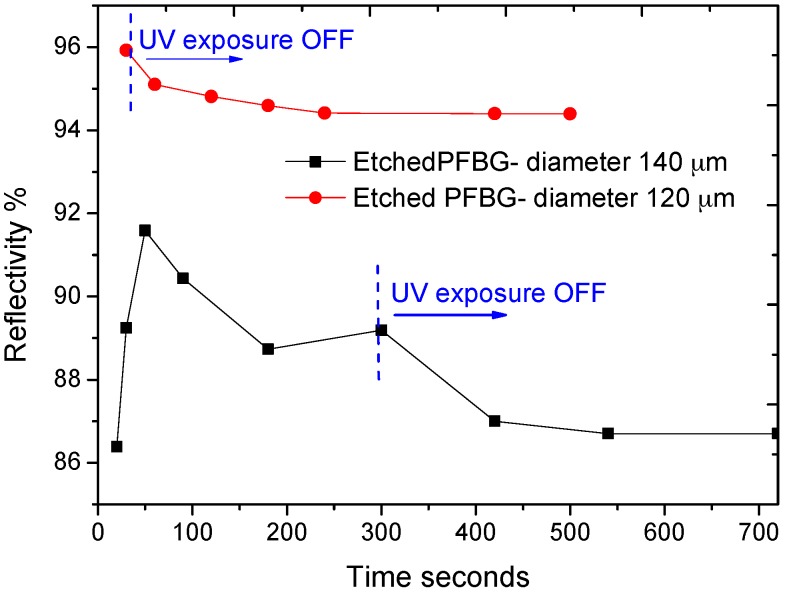
Evolved reflectivity of the etched PFBGs when the exposure is OFF at peak reflectivity and exposure is OFF after 5 min.

Short-term and long-term stability of the etched PFBGs are also crucial for the development of sensing devices for engineering applications. To study the change in reflectivity and the time required to attain stability, two scenarios were tested. In the first scenario, the immediate drop in reflectivity was measured just after the UV exposure is stopped, while in the second case the change in reflectivity was monitored after 5 min of continuous UV exposure even after obtaining peak reflectivity. As an example, changes in reflectivity of gratings inscribed in etched fibre with diameters of 140 µm and 120 µm are shown in Figure 4. In the first case where the exposure was stopped after obtaining peak reflectivity, a reduction of 5.34% was observed for the 140-µm fibre, whereas in the second case where the exposure was stopped after 5 min, the reflectivity dropped only 1.59% for the 120-µm fibre. In both cases, the reflectivity was stabilized after 500 s of UV exposure stoppage. Therefore, there is a trade-off between the UV exposure time and the time taken for the grating to attain stable reflectivity. The change in reflectivity is due to the residual photo polymerization activities occurring in the polymer fibre.

**Figure 5:**
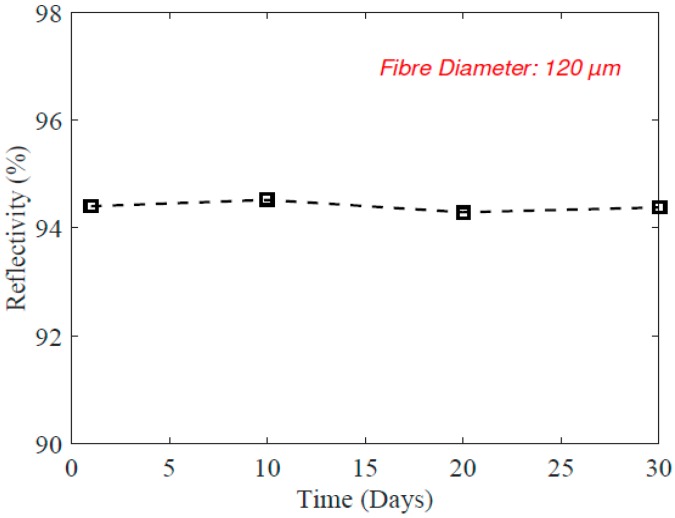
Measured reflectivity of the etched PFBGs for a period of 30 days.

The long-term stability of the etched PFBG was also studied. In this case, a 120-µm etched PFBG was used; the reflection spectrum was monitored for a month and is shown in Figure 5. It can be seen that the reflectivity remains constant, which indicates long-term optical power stability of the reflected signal. This test was carried out in an indoor laboratory atmosphere (room temperature ~20 °C, humidity ~45%). Further studies might be required for outdoor atmosphere to evaluate the influence of direct sunlight (UV content) and other changes in physical conditions such as temperature and humidity on the reflectivity and durability of the PFBG sensor.

## 4. Characteristic Properties of Etched Polymer Fibre Bragg Gratings

Characteristic properties of the etched PFBG that will have a direct influence on E and TEC were studied. The reduction in E and increase in TEC can positively influence the sensitivity of PFBG and thus it can be established that solvent etching of polymer fibre is one of the simplest ways to intrinsically enhance the sensitivity of Bragg gratings inscribed in such fibres.

### 4.1. Static Strain, Temperature, and Pressure

We have previously reported the strain, temperature and pressure sensitivity of unetched and etched PFBGs [10,11], which are summarized in this section. The enhanced sensitivity of these parameters is important in the design and development of biomedical sensors. In particular, the linearity of the response that allows the end user to select appropriate sensitivity is of critical importance.

**Figure 6:**
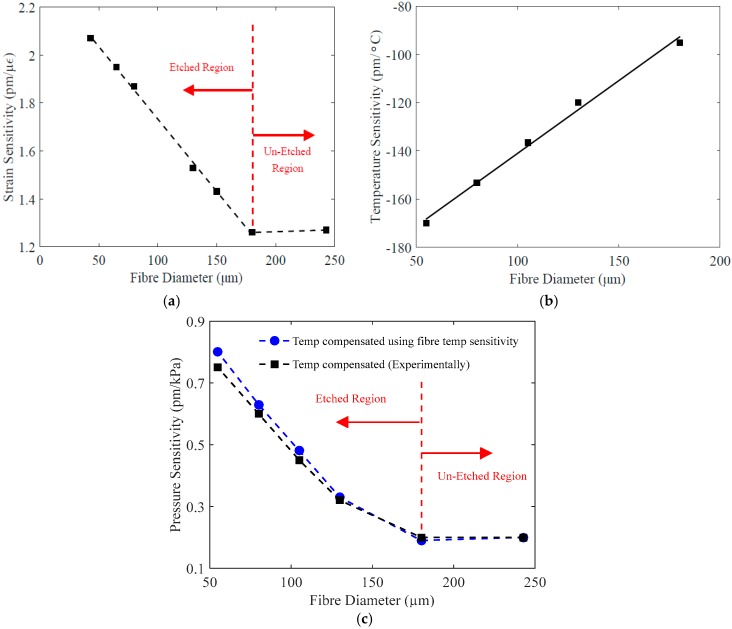
Sensitivity of the PFBG with different fibre diameters. (**a**) Strain; (**b**) temperature; (**c**) pressure.

The experimentally measured static strain sensitivity of etched and unetched PFBGs is shown in Figure 6a. The strain sensitivity of unetched PFBG was measured as 1.26 pm/µε and was confirmed with Bragg gratings inscribed in unetched fibres with diameters of 180 µm and 250 µm, both showing the same sensitivity and thereby confirming that fibre diameter reduction itself will not give enhanced sensitivity. A surface modification process such as etching was required to enhance the sensitivity. The etched PFBG with a diameter of 43 µm shows a sensitivity of 2.07 pm/µε, which is 64.2% higher than that of an unetched PFBG. Also, it can be seen in Figure 6a that the strain sensitivity increases linearly with decrease in etched fibre diameter, which means that the desired strain sensitivity for the PFBG can be obtained in a straightforward approach.

The measured temperature sensitivity of the etched and unetched PFBG is shown in Figure 6b. The unetched PFBG (180 µm) exhibits a temperature sensitivity of −95 pm/°C, while a 55 µm etched PFBG exhibits a temperature sensitivity of −170 pm/°C, which is 80% higher than that of the unetched PFBG. This considerable increase in the temperature sensitivity is correlated to the change in TEC of the material, which is proportional to the change in E. From Figure 6b it also is clear than the increase in temperature is linear with respect to reduction in fibre diameter. From Figure 6b, the change in temperature sensitivity per micro metre change in fibre diameter is 0.6 pm/°C. This linear behaviour will enable us to predict the temperature sensitivity of etched PFBG for any fibre diameter, if the sensitivity of unetched PFBG is known.

To measure the true hydrostatic pressure sensitivity, the effect of temperature has to be eliminated, as in a pressure chamber as the pressure varies the temperature also varies. Two methods were adopted for this. In the first case the etched PFBG was immersed in a highly viscous fluid within a pressure chamber (a sealed steel tube with optical fibre insertion option and fitted with a pressure gauge and connected to a gas cylinder with compressed air) where heat dissipation is minimal, and in the second case, the change in temperature was monitored while applying pressure (while pumping the air) and was compensated. The applied pressure range was from 0 kPa to 1000 kPa in 100-kPa steps. The obtained experimental sensitivity results by both methods are shown in Figure 6c. A pressure sensitivity of 0.75 pm/kPa was obtained for a PFBG with a diameter of 55 µm, which is an increase of 275% compared to unetched PFBG, which exhibits a pressure sensitivity of only 0.2 pm/kPa. Thus, it can be seen that the pressure sensitivity of PFBG has considerably increased, with a reduction in the fibre diameter via etching.

### 4.2. Hysteresis Characteristics

**Figure 7:**
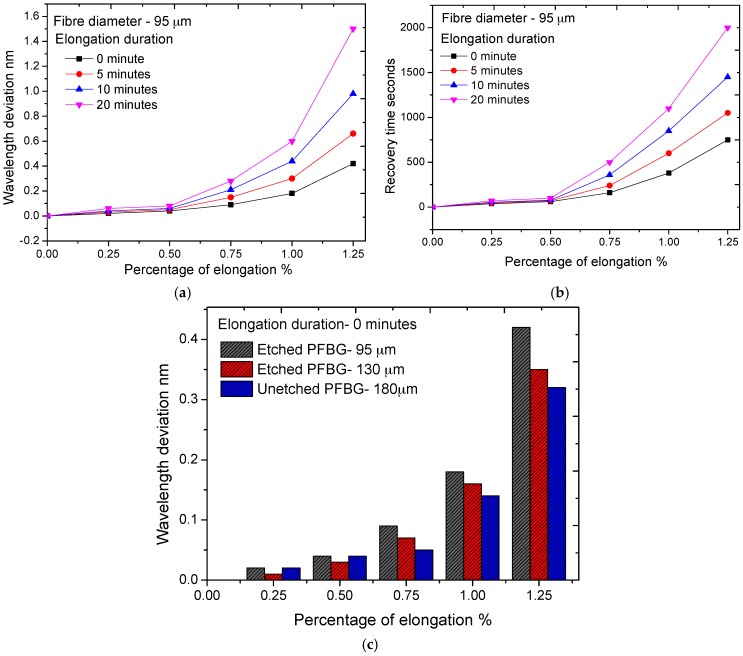
Effect of hysteresis on etched PFBGs for different elongations and time duration. (**a**) Measured wavelength deviation; (**b**) recovery time; (**c**) wavelength deviation of PFBG with different diameters.

To observe the hysteresis effect on etched PFBGs, axial elongation up to 1.25% was applied to an etched PFBG with a diameter of 95 µm using a precision translation setup where both ends of the fibre containing the grating were rigidly fixed. The elongation was applied at steps of 0.25% and to also study the effect of elongation duration on the hysteresis, the PFBGs were kept elongated for different time durations. The measured wavelength deviations for 0%–1.25% elongation for 0, 5, 10, and 20 min durations are shown in Figure 7a. It can be seen that the hysteresis in PFBG is highly time-dependent. For a 20 min duration, a wavelength deviation of 1.5 nm was observed, while it was only 0.35 nm for instant elongation and release (0 min). The recovery time of the etched PFBG (95 µm) was also measured and is presented in Figure 7b. Recovery time also depends on the applied elongation and duration. For a 1.25% elongation applied for 20 min, it required more than 33 min (2000 s) to recover to its original wavelength (strain), while for an applied elongation of 0.25% with zero duration it was only 38 s. The observed hysteresis and recovery time are also fibre diameter-dependent and increase as the fibre diameter reduces. Figure 7c shows hysteresis effect of unetched (180 µm) and etched PFBGs with different diameters (130 µm and 95 µm) for different elongations for zero duration. From the results it can noted that, though etched PFBG has high intrinsic strain, temperature and pressure sensitivity, it suffers from the hysteresis issue, especially at higher strain levels, and therefore the working range should be limited to lower strain levels to eliminate the effect of hysteresis. For elongation of less than 0.25% (2500 µε) the hysteresis is considerably less (wavelength deviation less than 0.02 nm) and is similar across all fibre diameters as seen Figure 7c. Therefore, for high-end applications such as in biomedical sensing, by further limiting the elongation to 0.05% (500 µε), the effect of hysteresis can be eliminated to a great extent.

### 4.3. Dynamic Strain Characteristics

The dynamic strain sensitivity of the etched and unetched PFBGs up to a frequency of 1 kHz was also studied. To date, due to the lack of a high frequency optical fibre measurement system compatible with PFBGs, the dynamic properties of PFBGs were unknown. As etched PFBGs can improve the reflectivity and sensitivity, they can be used with commercial dynamic FBG interrogators, such as IMON-256 (Ibsen Photonics A/S, Farum, Denmark) which was used in this study. The impact of etching and modified material parameters on the fibre relaxation was investigated to determine the actual frequency range and dynamic sensitivity of the etched PFBG towards high-frequency strain variations.

**Figure 8:**
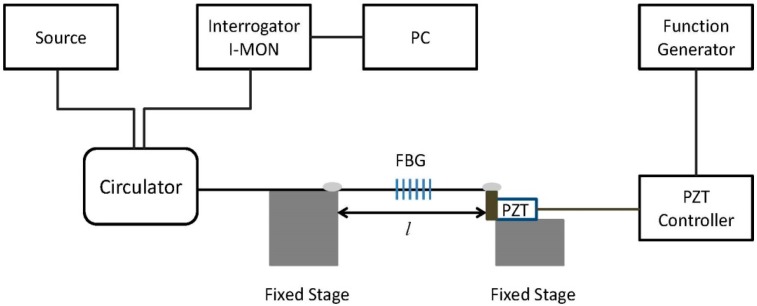
Schematic of experimental set-up for dynamic strain characterization. PZT: piezo electric transducer. PC: personal computer.

To observe the dynamic properties, an experiment was carried out for an unetched PFBG with a diameter of 180 µm and an etched PFBG with a diameter of 130 µm. The experimental setup for dynamic strain sensing is shown in Figure 8, where a piezo electric transducer (PZT) (model AE0505D18F, Thorlabs, Newton, NJ, USA) controlled by a PZT controller (model MDT694B, Thorlabs, Newton, NJ, USA) and function generator was used to apply the dynamic frequencies up to 1 kHz.

**Figure 9:**
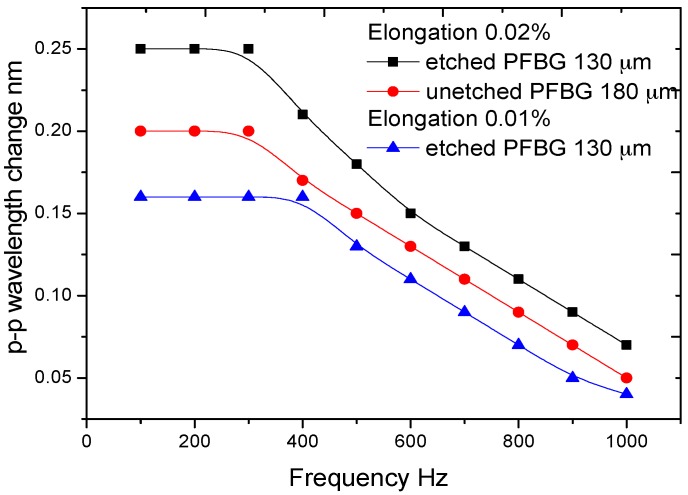
Dynamic strain characteristics of etched and unetched PFBGs.

To eliminate the hysteresis issues, a dynamic elongation of 0.02% (200 µε) was applied to the PFBGs and the peak-to-peak changes in the wavelength were measured and are shown in Figure 9. The frequency dependencies of the PZT and the measurement system were compensated, and Figure 9 shows the true dynamic behaviour of the PFBGs. At 0.02% elongation it can be seen that the frequency-independent responses range from 0 to 300 Hz for both the PFBGs, while the etched PFBG shows high wavelength sensitivity in this frequency range compared to the unetched PFBG. The p-p wavelength change decreases thereafter, and at 1 kHz the etched PFBG shows a p-p wavelength change of 0.07 nm compared to 0.05 for the unetched PFBG. To further study the effect of applied elongation/strain on the dynamic range of the etched PFBGs, an elongation of 0.01% (100 µε) was applied to the etched PFBG and was compared to that of 0.02%. This is also shown in Figure 9. It can be seen that for 0.01% elongation a flat frequency response was obtained up to 400 Hz. The 3 dB frequencies of the etched PFBG were 650 Hz and 550 Hz for 0.01% and 0.02% elongation, respectively, while the value was 500 Hz for the unetched PFBG with 0.02% elongation. This indicates that etched PFBG can have a larger dynamic range than the unetched PFBG, and by further reducing the fibre diameter the dynamic range can be broadened. The result also indicates that there is a trade-off between wavelength sensitivity and frequency range of the PFBG.

## 5. Biomedical Applications of the High-Sensitivity Etched PFBG Sensor

One of the areas where PFBGs can be useful compared to silica fibres is in biomedical/biomechanical applications, due their less fragile nature. The enhanced sensitivity and long-term stability of etched PFBGs would further increase their suitability in biomedical applications. Two sensing devices based on etched PFBG for biomedical applications are demonstrated.

### 5.1. Blood Pressure Transducer—Design and Experimental Tests

**Figure 10:**
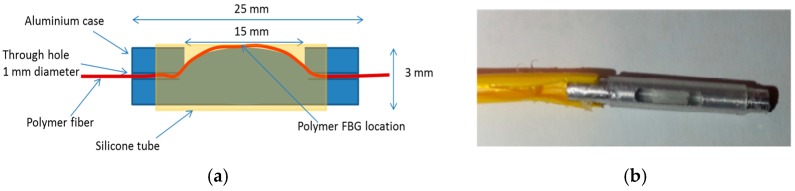
(**a**) Design of the pressure transducer; (**b**) developed prototype of the transducer.

In this demonstrated example, a pressure transducer was designed and fabricated with an etched fibre diameter of 150 µm. The transducer design is shown in Figure 10a and the prototype of the transducer is shown in Figure 10b. An aluminium cylinder (3 mm × 25 mm) was used as the body and is machined to create a path to insert the PFBG and to apply pressure to the fibre as shown in Figure 10a. After inserting the fibre, the ends of the tube were glued to keep the fibre secure. A silicone tube was then inserted on to the cylinder which acts as a diaphragm that transfers pressure to the PFBG underneath. When pressure is exerted on to the silicone diaphragm, compressive strain will be exhibited on the PFBG and as a result there will be negative wavelength shift for the peak reflected signal.

**Figure 11:**
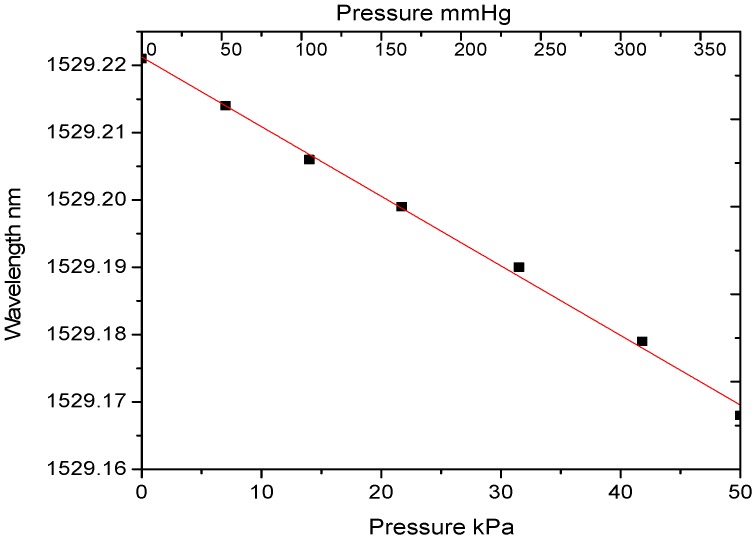
Pressure vs. wavelength change of the PFBG pressure transducer.

To characterize the pressure transducer, it was placed in a chamber and pressure was exerted using an air pump. It was also monitored using a reference pressure transducer (Testo 312-3). The reference pressure transducer has a measuring range of −600 kPa to +600 kPa, with a resolution of 0.1 kPa and an accuracy of ±0.4 kPa. The wavelength change (temperature compensated) corresponding to the applied pressure measured using the reference pressure transducer is shown in Figure 11. As the pressure transducer was intended for blood pressure measurements, a pressure range from 0 to 375 mmHg (0–50 kPa) was applied. In this pressure range the effect of hysteresis is also minimal as discussed in Section 4.2. The demonstrated results indicate a pressure sensitivity of 1.2 pm/kPa. This can be further improved by reducing the fibre diameter or by altering the transducer design.

### 5.2. Breathing Rate Monitoring Device

**Figure 12:**
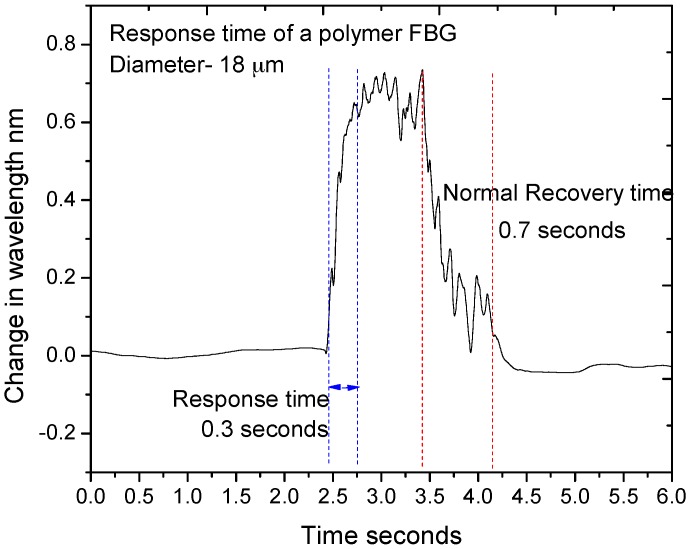
Response and recovery time of the 18 µm PFBG.

The high temperature sensitivity and fast response time of the etched PFBG allow it to be used as a breathing rate monitoring device. The fibre was etched to a smaller diameter of 18 µm to increase the temperature sensitivity and to reduce the response time. In order to measure the response time (time taken to observe 10–90% change) of the 18-µm diameter PFBG, a sudden burst of hot air was blown onto the PFBG and the change in the wavelength response was measured and is shown in Figure 12. It can be seen from the figure that a response of time of 0.3 s and a recovery time of 0.7 s was observed. Considering this, the measurable breathing rate of the sensor would be limited to ~0.8–1 Hz. Given this limitation, a breathing rate up to 48–60 breaths per minute can be measured using the 18-µm diameter etched PFBG. However most of the human breathing rates are still within this range and the typical adult breathing rate is 12–20 breaths/min.

**Figure 13:**
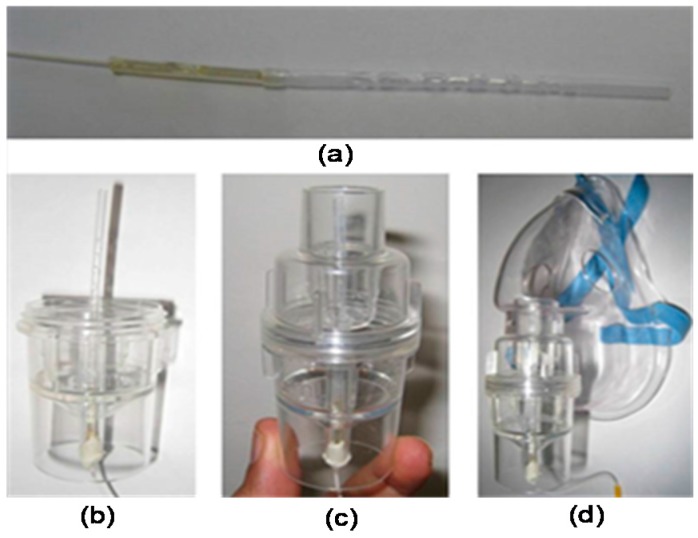
Etched PFBG breath rate monitoring setup. (**a**) Packaged sensor; (**b**–**d**) the sensor attached to a nebulizer mask.

**Figure 14:**
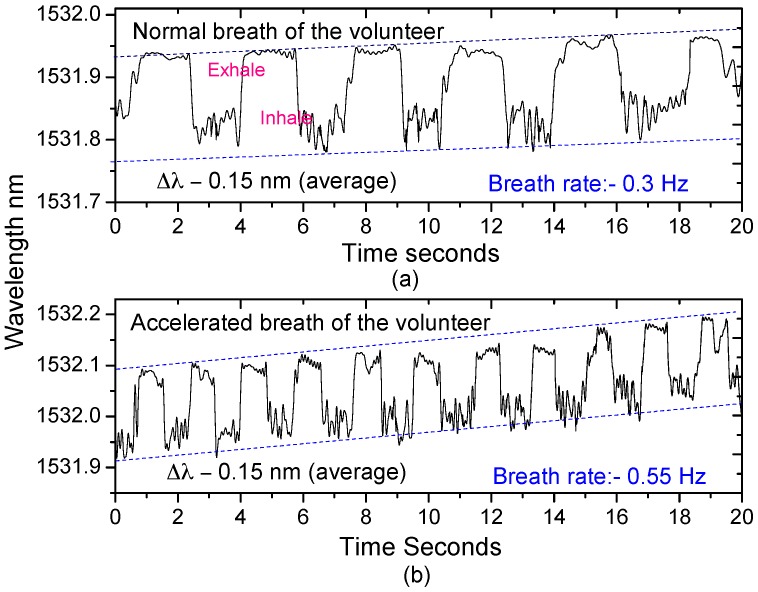
Breathing pattern of a volunteer measured by the etched PFBG sensor. (**a**) Normal breathing; (**b**) accelerated breathing.

To test the PFBG to monitor breathing, an 18 µm etched PFBG was packaged into a plastic tube with a diameter of 3 mm and length of 60 mm with a number of holes, where breathing air can easily be circulated around the PFBG as shown in Figure 13a. The packaged sensor was inserted into a nebulizer mask as shown in Figure 13b–d for breathing rate monitoring. The normal and accelerated breathing rate of a volunteer was measured using the packaged sensor and is shown in Figure 14. A breathing rate of ~0.3 Hz was measured (Figure 14a), which is within the range for a human adult. An accelerated breathing rate was also measured and is shown in Figure 14b. The change in wavelength observed during the breath process is ~0.15 nm, which arises from the change in temperature and humidity conditions.

The long-term drift as seen in Figure 14 arises from the change in humidity which the PFBG is highly sensitive to, and is also due to residual temperature change. Data in Figure 14 was raw data with no signal processing or filtering applied. However, as the breathing is a dynamic process, by applying proper signal processing and filtering, the static effects can be eliminated.

Though only two applications are demonstrated here, given the high sensitivity of etched PFBG sensors it has the potential to be used in applications such as medical textiles and wearables [15].

## 6. Conclusions

Fabrication of etched PFBGs and their characteristic properties and potential applications were discussed. A solvent etching method was adopted, and due to the etching of the material, the mechanical properties of the polymer fibre were modified, which resulted in a unique way to intrinsically enhance the sensitivity of gratings inscribed in etched polymer fibres. Grating growth characteristics, stability, and characteristic properties such as temperature, static and pressure were also discussed. The results showed that the etched PFBG sensors showed higher intrinsic sensitivity compared to unetched ones. As long-term stability, hysteresis, and dynamic strain sensitivity are of importance to biomedical applications, those characteristics were also studied. To demonstrate the capability of the etched PFBGs in biomedical applications where high sensitivity is required, a blood pressure transducer and a breathing rate monitoring device were designed and demonstrated to measure pressure in the blood pressure range and measure human breathing rate, respectively, in different conditions. It is anticipated that the results presented in this paper will start a new research direction in polymer fibre Bragg gratings and the development of high sensitivity sensors based on them.
